# Better maternity care pathways in pregnancies after stillbirth or neonatal death: a feasibility study

**DOI:** 10.1186/s12884-022-04925-3

**Published:** 2022-08-10

**Authors:** Tracey A. Mills, Stephen A. Roberts, Elizabeth Camacho, Alexander E. P. Heazell, Rachael N. Massey, Cathie Melvin, Rachel Newport, Debbie M. Smith, Claire O. Storey, Wendy Taylor, Tina Lavender

**Affiliations:** 1 Department of International Public Health, Centre for Childbirth, Women’s and Newborn Health, Liverpool School of Tropical Medicine. Pembroke Place, Liverpool, L3 5QA UK; 2 Division of Population Health, Health Services Research and Primary Care, School of Health Sciences, The University of Manchester, Oxford Rd, Manchester, M13 9PL UK; 3 Division of Developmental Biology and Medicine, School of Medical Sciences, The University of Manchester, Manchester, M13 9PL UK; 4 East Lancashire Hospitals NHS Trust, Royal Blackburn Hospital, Blackburn, BB2 3HH England; 5 Northern Care Alliance NHS Trust, Royal Oldham Hospital, Oldham, OL1 2JH England; 6 Division of Psychology and Mental Health, Manchester Centre for Health Psychology, School of Health Sciences, The University of Manchester, Oxford Rd, Manchester, M13 9PL UK; 7 Patient and Public Involvement Investigator, Bristol, UK; 8 Division of Nursing Midwifery and Social Work, School of Health Sciences, The University of Manchester, Oxford Rd, Manchester, M13 9PL UK

**Keywords:** Pregnancy, Stillbirth, Neonatal death, Feasibility study, Antenatal care, Maternity experiences

## Abstract

**Background:**

Around 1 in 150 babies are stillborn or die in the first month of life in the UK. Most women conceive again, and subsequent pregnancies are often characterised by feelings of stress and anxiety, persisting beyond the birth. Psychological distress increases the risk of poor pregnancy outcomes and longer-term parenting difficulties. Appropriate emotional support in subsequent pregnancies is key to ensure the wellbeing of women and families. Substantial variability in existing care has been reported, including fragmentation and poor communication. A new care package improving midwifery continuity and access to emotional support during subsequent pregnancy could improve outcomes. However, no study has assessed the feasibility of a full-scale trial to test effectiveness in improving outcomes and cost-effectiveness for the National Health Service (NHS).

**Methods:**

A prospective, mixed-methods pre-and post-cohort study, in two Northwest England Maternity Units. Thirty-eight women, (≤ 20 weeks’ gestation, with a previous stillbirth, or neonatal death) were offered the study intervention (allocation of a named midwife care coordinator and access to group and online support). Sixteen women receiving usual care were recruited in the 6 months preceding implementation of the intervention. Outcome data were collected at 2 antenatal and 1 postnatal visit(s). Qualitative interviews captured experiences of care and research processes with women (*n* = 20), partners (*n* = 5), and midwives (*n* = 8).

**Results:**

Overall recruitment was 90% of target, and 77% of women completed the study. A diverse sample reflected the local population, but non-English speaking was a barrier to participation. Study processes and data collection methods were acceptable. Those who received increased midwifery continuity valued the relationship with the care coordinator and perceived positive impacts on pregnancy experiences. However, the anticipated increase in antenatal continuity for direct midwife contacts was not observed for the intervention group. Take-up of in-person support groups was also limited.

**Conclusions:**

Women and partners welcomed the opportunity to participate in research. Continuity of midwifery care was supported as a beneficial strategy to improve care and support in pregnancy after the death of a baby by both parents and professionals. Important barriers to implementation included changes in leadership, service pressures and competing priorities.

**Trial registration:**

ISRCTN17447733 first registration 13/02/2018.

**Supplementary Information:**

The online version contains supplementary material available at 10.1186/s12884-022-04925-3.

## Background

Despite encouraging reductions in rates for England and Wales in recent years, 4,090 babies were stillborn or died in the first 28 days after birth during 2020 [1]. The death of a baby before, during or soon after birth is acknowledged as amongst the most traumatic life-events for parents [2]. Most women will conceive again, and subsequent pregnancies are often characterised by increased anxiety, stress and emotional vulnerability, particularly when inter-pregnancy intervals are short [3–5]. Psychological distress in pregnancy is associated with adverse outcomes and after perinatal death often persists beyond the birth of a healthy child, risking disrupted maternal-infant attachment and parenting difficulties [6]. A metasynthesis of studies of parents’ experiences has confirmed this emotional flux and doubts regarding the likelihood of a positive outcome in subsequent pregnancy [7]. Moreover, psychological distress often led to tensions in close personal and family relationships. Resulting social isolation increased parent’s reliance on health professionals and confirmed the importance of appropriate emotional and psychological support from caregivers during pregnancy.

Our previous national survey of 546 women who had experienced pregnancy after stillbirth or neonatal death, across all UK regions and > 60% of NHS maternity units demonstrated a lack of equity in current provision [8]. Although some excellent care was identified; negative experiences were common. Poor communication, insensitive comments and unawareness of parents’ previous history and perceived lack of empathy reflected other studies [9, 10]. Organisational factors and service delivery were also prominent including lack of relational continuity; parents rarely saw the same professionals at their appointments and often had to recount details of their previous baby’s death on several occasions. Fragmented care in standard high-risk obstetric models, where parents encountered multiple professionals, was linked to inadequate emotional support. The relationship between the mother/ parents and the midwife, who has the most direct and intimate contacts with the family is recognised as, perhaps, the most important influence on perceptions of emotional support and quality [11]. Therefore, the impact of improved continuity of midwifery care has been an important focus for research. In both low and high-risk pregnancies, midwife-led and case-holding models reduce interventions, are safe, cost-effective and improve maternal satisfaction compared to standard care [12–14]. However, continuity models had not been implemented widely in UK care, national survey of 23,000 women in 2013 reported that only 34% of women saw the same midwife for most or all antenatal visits [15].

In pregnancies after stillbirth or neonatal death women and families, arguably have a greater need for intensive midwifery support and increased relational continuity could address many of the shortcomings identified in existing services. The Midwifery 2020 report [16] identified the role of the midwife as ‘coordinator of care’ for women with complex pregnancies, emphasising the pivotal role for a known midwife ‘in coordinating the journey through pregnancy ensuring that… holistic care is provided to optimise each woman’s birth experience regardless of risk factors.’ (pg. 23); but provided little guidance on how this would be facilitated in practice. The UK National Maternity Review (2016) renewed impetus for more personalised, responsive care, including midwifery continuity [17]. The study utilised ‘care co-ordination’, which has been valuable in other healthcare settings, in an intervention to increase relational continuity in the antenatal period combined with access to additional support, designed to address shortcomings in existing care during subsequent pregnancy. Here, we aimed to explore the feasibility of implementation of the package of support and of a full-scale trial to test effectiveness for women in pregnancy after stillbirth or neonatal death.

## Methods

### Design and study setting

Following the MRC/NIHR framework for development and evaluation of complex interventions in health care [18], a prospective, mixed-methods feasibility study, using a ‘pre’ and ‘post’ observational design was conducted in two NHS maternity units in the North-West of England, from March 2018 to July 2020. Sites were large, urban obstetric units in areas of significant ethnic and social diversity. Research governance approvals were confirmed by NW Greater Manchester West Research Ethics Committee (18/NW/0010), and the study prospectively registered (ISRCTN17447733). Patient and public involvement (PPI) was embedded throughout the research process, CS a bereaved parent and International Stillbirth Alliance (ISA) board member was a co-investigator. Additional input was provided by bereaved parents with experience of subsequent pregnancy, during design, prior to seeking funding and throughout study conduct and interpretation of findings by parent members of the Technical Advisory Group (TAG).

### Study intervention

The intervention was co-designed with bereaved parents, midwives and service managers at the research sites, and informed by previous exploratory work and a literature review [7, 8]. Women who were pregnant after stillbirth or neonatal death and recruited during the intervention period were allocated a midwife care-coordinator, a registered midwife (hospital or community at the included site) with previous experience of caring for bereaved parents and study-specific training. Care coordinators were supported by a ‘buddy’ (2^nd^ named midwife) to cover for leave and any other absences. The proposed care coordinator role is outlined in Table 1. In addition, monthly in-person support group sessions, facilitated by the research team were scheduled at each site, and a study ‘WhatsApp’ messaging group offered.

**Table 1:** Care coordinator proposed role

When	What
Recruitment (≤ 20 weeks’ gestation)	• Meet with woman (and partner or birth partner) • With woman and lead obstetrician, devise/ review care plan to include schedule of visits, monitoring any additional investigation
Antenatal contacts	• Provide midwifery care, where possible, during scheduled antenatal visits • If woman having additional appointments/investigations e.g., medical clinics, liaise with multidisciplinary professionals, departments to ensure effective communication • Be available for non-urgent contacts, maintain regular contact (by woman’s preferred method SMS, call, email, e.g., 1–2 weekly to ascertain need for further support)
Intrapartum care plan	• Initiate discussion/planning of intrapartum care determine individual needs and preferences. Written plan in notes, visit labour ward, introductions to staff
Postnatal	• Make contact within 72 h of birth, final contact before transfer to (primary care) health visitor^a^

^a^ Specialist public health nurse, who supports families with children under 5 in UK

### Study sample and recruitment

The planned sample was 60 pregnant women ≥ 16 years, who had experienced the stillbirth or neonatal death of any previous baby and booked for care in the included sites. Women were also required to be ≤ 20 weeks pregnant at recruitment and not previously referred to an existing medical/obstetric clinical service (e.g., cardiac disease, diabetes clinics). Additionally, participants were required to have sufficient command of English to complete study questionnaires and qualitative interviews without the assistance of a translator. The sample size was determined using published guidance for feasibility studies and was considered sufficient to allow implementation of the study intervention in two sites and assessment of the feasibility of a full scale trial [19]. During the first phase of the study (pre-intervention; March to August 2018), we aimed to recruit 20 women (10 per site) offered the usual pattern of care according to their clinical needs; during the second phase (intervention; Sept 2018-Sept 2019), a further 40 women would be recruited (20 per site) and offered the study intervention, in addition to usual care. Prior to the study, workshops were held for clinical staff at all participating sites to inform them about the research. Eligible women were identified via antenatal clinical care teams, who introduced the study. Women who were interested in receiving further information provided written ‘consent for contact’ by the research midwife. This was followed by a detailed verbal explanation, supported by the participant information sheet (PIS), opportunities for questions and time to consider. Informed consent was then confirmed in writing. Domestic, or birth partners (family members or friends, identified as the primary support during pregnancy by the woman herself) were recruited, via the woman, to explore experiences via questionnaires (domestic partners only) and/or qualitative interviews. Unwillingness or unavailability of a partner or birth partner did not affect the woman’s eligibility. After the second phase was completed, health workers (the potential sample included midwives, obstetricians, and service managers) involved in the delivery of the intervention were also recruited for qualitative interviews.

### Outcome measures

The primary outcome for feasibility was recruitment and retention of women and their partners in the study; a trial would be considered feasible if recruitment targets were met and ≥ 75% of women participants were retained until completion. Secondary outcomes included experiences of study participation and the intervention, amongst women, partners (or birth partners) and health workers, to determine the acceptability of trial processes and of the intervention. The feasibility of data collection and characteristics of the proposed psychological, cost-effectiveness, utility, and clinical outcome measures, along with process outcomes including quality of implementation were also assessed.

### Data collection

**Fig. 1:**
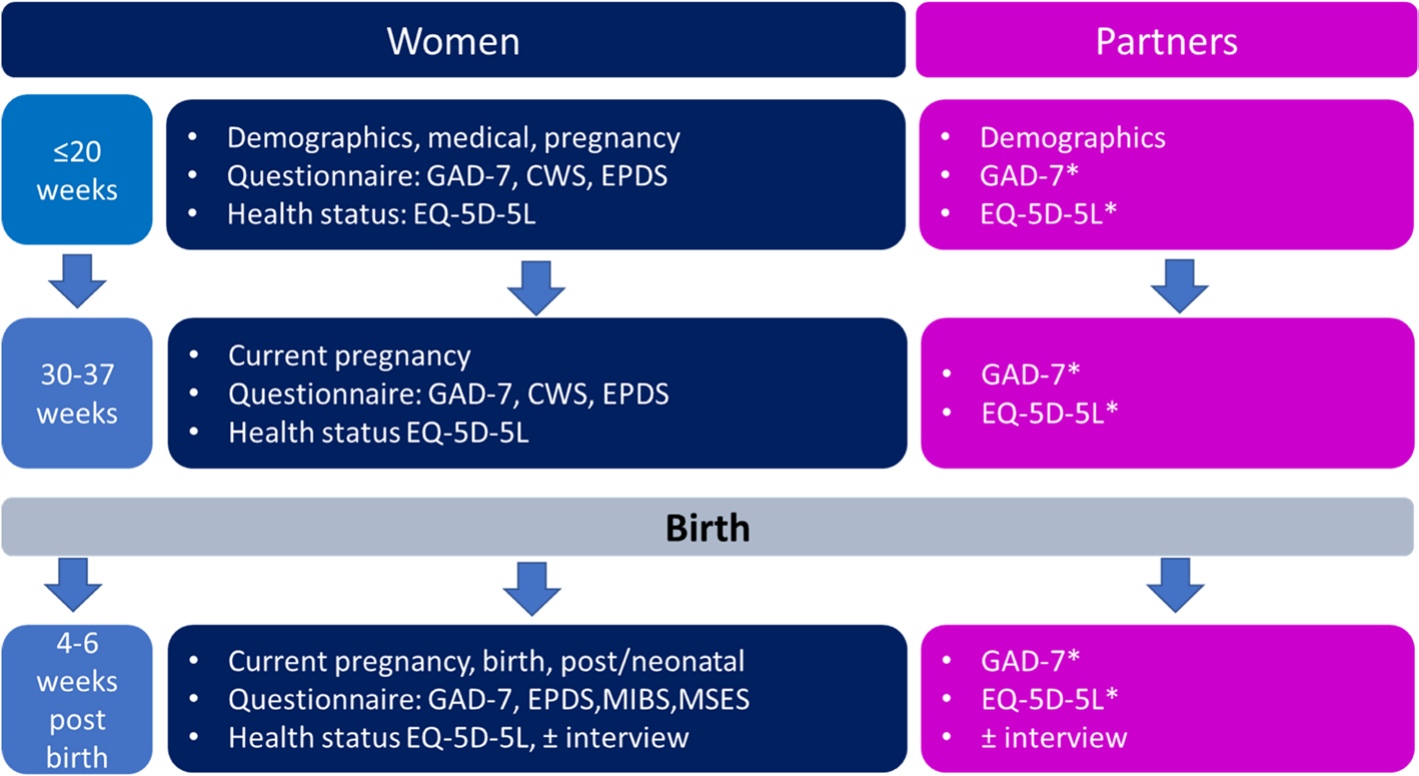
Data collection process. Legend: GAD-7 Generalised anxiety disorder assessment – 7 item, CWS: Cambridge Worry Scale, EPDS Edinburgh postnatal depression scale, MIBS: Maternal infant bonding scale, MSES: Maternal self-efficacy scale. *Questionnaires were completed by domestic partners only; interviews included domestic partners and birth partners

Data collection processes are outlined in Fig. 1. Participant data from women and domestic partners was collected at three meetings with the research midwife: At recruitment (≤ 20 weeks’ gestation), in late pregnancy (30–37 weeks’) and postnatally, 4–6 weeks after birth of the baby.

Investigator-designed case report forms (researcher-administered, demographic, pregnancy, clinical data including health care utilisation) and questionnaires (self-report; validated psychological and health utility measures) were used. Psychological measures included the Generalised Anxiety Disorder 7-item scale (GAD-7) [20], Cambridge Worry Scale (CWS) [21], Edinburgh Postnatal Depression Scale (EPDS) [22], Maternal Infant Bonding Scale (MIBS) [23], Maternal Self Efficacy Scale (MSES) [24]. The EQ-5D-5L is a 5 dimension, descriptive system for measuring health utility and quality of life in adults [25]. Semi-structured qualitative interviews were conducted by two researchers (TM, WT) with a subsample of women, and separately with partners in both phases, 4–6 weeks after the birth. Midwives, including care coordinators involved in intervention delivery were interviewed after completion of phase 2. Interviews were held in a place of the participant’s choice. Before March 2020, women and partners were interviewed at their homes. After the onset of COVID-19, remaining data collection (for 2 participants) and all health worker interviews were conducted remotely via telephone. Interviews were digitally audio-recorded and transcribed verbatim. Women participants were also provided with a paper diary to allow contemporaneous recording of thoughts and feelings around pregnancy and their care. An intervention log, completed by the care coordinator and research midwife after contacts, captured implementation of intervention components, including frequency and any issues encountered.

### Data analysis

Case report forms and questionnaires were verified for completeness and accuracy by a second researcher, before input into a custom-designed SPSS database. Outcome measures were compared descriptively using frequencies and percentages, descriptive statistics including mean and standard deviations, median and ranges for numerical variables. Data from psychological tools were evaluated to determine whether characteristics were comparable across different measures. Health utility was derived from the EQ-5D-5L using the crosswalk mapping approach (as recommended by NICE at the time of analysis) and reported as quality-adjusted life years [QALYs]) [26]. The cost of delivering the intervention was estimated based on the number of hours of training received and care delivered by care coordinators, and the 2019 unit cost per hour of their time (£47/hour) [27]. The cost of the senior midwife (£56/hour) and health psychologist (£56/hour) to deliver the training was also included.

Qualitative data (interviews, logs and diaries) were analysed using an inductive approach, following the six recursive phases of thematic analysis by WT, RM and TM [28]. Analysis uncovered experiences of care in subsequent pregnancies, participation in the research including recruitment processes, the acceptability and implementation of the intervention and burden of data collection. Interpretation of the qualitative findings were discussed and agreed by the wider research team.

## Results

### Recruitment and retention

**Fig. 2:**
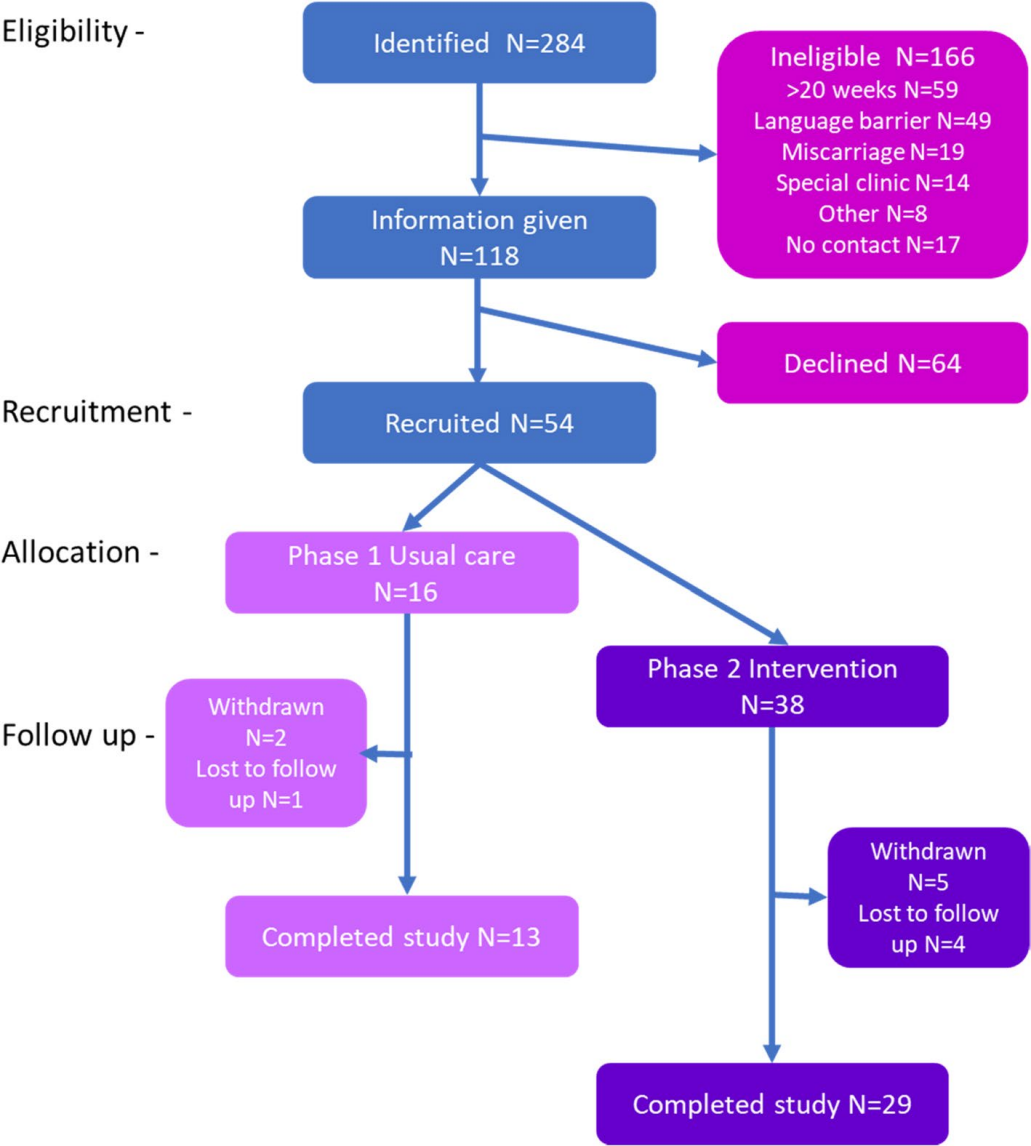
Participant flow

Recruitment log data were used to assess the willingness of women and partners to participate and continue in the research (Table 2). Participant flow for the study is summarised in Fig. 2. In site 2, initial recruitment was below target due to fewer eligible participants being available than anticipated, mainly due to language issues. By the end of Phase 1, 16 women (80% of the planned sample) and 10 partners were recruited. This was judged sufficient to meet the objectives therefore, Phase 2 recruitment commenced as planned. In Phase 2, unexpectedly low numbers of eligible women were identified across both sites in November and December 2018, therefore recruitment was extended until November 2019 (14 months vs. 12 months planned). In Phase 2, 38 women (95% of the planned sample) and 14 partners were recruited.

**Table 2:** Recruitment and retention

Recruitment	Site 1	Site 2	Total
Identified (% total)	131 (46%)	153(54%)	284
Not eligible (% identified)	68 (52%)	98(64%)	166 (58%)
Reasons not eligible (% identified)			
> 20 weeks	21 (16%)	38 (25%)	59 (21%)
Language barrier	13 (10%)	36 (23%)	49 (17%)
Unable to contact	13 (10%)	4 (3%)	17 (6%)
Miscarriage	10 (8%)	9 (6%)	19 (7%)
In specialist clinic	7 (5%)	7 (5%)	14 (5%)
Other	4 (3%)	4(2%)	8 (3%)
Given information	63	55	118
Recruited (% received information)	32 (51%)	22 (40%)	54 (46%)
Declined	31 (49%)	33 (60%)	64 (54%)
Main reason (% declined)			
Unwilling to commit	24 (77%)	27 (81%)	51 (80%)
Unwilling to complete questionnaires	7 (23%)	6 (18%)	13 (20%)
Retention			
Completed study (% recruited)	25 (78%)	17 (77%)	42 (77%)
Withdrew (% recruited)	5 (16%)	3 (14%)	8 (15%)
Main reasons			
Unwilling to complete questionnaires	3	2	5
Adverse outcome	1 (2^nd^ trimester miscarriage)	1 (Preterm birth NND)	2
Moved	1	0	1
Lost to follow up	2 (postnatal)	2 (1 postnatal)	4 (7%)

### Recruitment rates

Of 284 women initially identified as potentially eligible, 118 (42%) were confirmed to meet the inclusion criteria. The main reasons for ineligibility were gestation over 20 weeks at identification (*N* = 59; 21% of total identified), a further 49 women (17%) were judged to lack sufficient command of English to complete data collection without a translation of materials or interpretation. Of the 118 women given study information, 54 (46%) agreed to participate, site 1 had a higher recruitment rate than site 2 (51% vs. 40%, respectively). Women declining to participate were invited to supply reasons to the research midwife. Most provided nonspecific responses; commonly that they did not want to commit to the research (80%), several women approached in both phases mentioned reluctance to attend research follow-up visits. A few women approached in the intervention phase did not want to engage with the study intervention. In both phases, a small number of women specifically expressed unwillingness to complete the required questionnaires (20%). Partners and birth partners were recruited via the women. All partners and birth partners who gave consent to be contacted, agreed to participate in the study. Where a partner or birth partner was not identified or did not wish to be contacted specific reasons were not sought.

### Participants

The characteristics of the women and partners/birth partners participating in the study are summarised in Tables 3 and 4. For women, age, body mass index, employment, and relationship status did not differ notably across the sites and phases, around 15% of women were current cigarette or e-cigarette smokers. A minority had degree-level (typically university Bachelors' degree; 6) education (Phase 1: 31% vs Phase 2: 29%) and reflecting the diversity of the communities in the settings, 41% of women described their ethnicity as Asian or Black. Regarding obstetric history, the number of previous pregnancies was similar; however, there were differences in the distribution of stillbirths versus neonatal deaths across phases. In Phase 1, 13 (81%) of included women had previously experienced a stillbirth, and 3 (19%) a neonatal death, whilst in Phase 2, 21 (55%) and 18 (47%) women had prior stillbirths and neonatal deaths, respectively. One woman (Phase 2) had experienced both a stillbirth and a neonatal death, previously. In Phase 1, 10 partners and one birth partner participated, in phase 2, 14 partners participated. Of 54 women recruited, 42 (77%) completed the study and similar rates were observed in both phases. Eight women (15%) withdrew from the study before the end of data collection; 5 indicated unwillingness to complete further questionnaires, and one moved out of the area. Two women withdrew after adverse outcomes, including a second trimester miscarriage and extremely pre-term birth followed by neonatal death and two women (7%) were lost to follow up in the postnatal period.

**Table 3:** Characteristics of pregnant women

Characteristics	Phase 1 (pre intervention)			Phase 2 (intervention)		
	Site 1	Site 2	Total	Site 1	Site 2	Total
N	14	2	16	18	20	38
Age: Median (range)	30 (20–37)	30 (28–31)	30 (20–37)	31 (22–35)	34 (27–46)	31 (22–46)
Ethnicity						
White	7 (50%)	1 (50%)	8 (50%)	10 (56%)	14 (70%)	24 (63%)
Asian	7 (50%)	1 (50%)	8 (50%)	8 (42%)	4 (20%)	12 (32%)
Black	0	0	0	0	2 (10%)	2 (2%)
Employment						
Yes	8 (57%)	2 (100%)	10 (62%)	10 (56%)	12 (63%)^a^	22 (59%)
Homemaker	4 (29%)	0	4 (25%)	0	0	0
No	2 (4%)	0	2 (12%)	8 (44%)	7 (37%)^a^	15 (41%)
Relationship						
Married/civil partner	9 (64%)	2 (100%)	11 (69%)	10(56%)	11 (55%)	21 (55%)
Partner	3 (21%)	0	3 (19%)	4 (22%)	7 (35%)	11 (29%)
Divorced	0	0	0	0	0	0
Single	1 (7%)	0	1 (6%)	4 (22%)	2 (10%)	6 (16%)
Highest Level Education						
Degree or above	4 (29%)	1 (50%)	5 (31%)	4 (22%)	7 (35%)	11 (29%)
A level equiv.	5 (36%)	1 (50%)	6 (38%)	5 (28%)	6 (30%)	11 (29%)
GCSE equiv.	2 (14%)	0	2 (12%)	7 (39%)	6 (30%)	13 (34%)
No quals	3 (21%)	0	3 (19%)	2 (11%)	1 (5%)	3 (8%)
BMI: Median (range) [Booking]	28 (17–45)	24 (24)	26 (17–45)	26 (19–32)	28 (20–38)^a^	27(19–38)
Current smoker (including e- cigarettes)	2 (14%)	0	2 (12%)	3 (18%)	3 (15%)^a^	6 (17%)
Current alcohol	0	0	0	0	0	0
No of previous pregnancies: Median (range)	2 (1–4)	2 (1–3)	2 (1–4)	3 (1–5)	4 (2–7)	3 (1–7)
Previous stillbirth	11 (79%)	2 (100%)	13 (81%)	12 (69%)	9 (45%)^b^	21 (55%)
Previous neonatal death	3 (19%)	0	3 (19%)	6 (33%)	12 (60%)^b^	18 (47%)
First pregnancy after SB/NND	6 (43%)	1 (50%)	7 (44%)	5 (28%)	0	5 (13%)
Gestation at first visit Median (range)	19 (12–21)	14 (9–20)	19 (9–21)	17 (11–21)	17 (13–24)	17 (1–24)

NB: ^a^ Missing data for 1 participant; ^b^ 1 participant experienced both a SB and a NND

**Table 4:** Characteristics of partners and birth partners

Characteristics	Phase 1	Phase 2
N	11 (site 1 = 9, site 2 = 2) ^b^	14 (site 1 = 8, site 2 = 6)
Age: Median (range)	36 (30–57)	33 (24–58)
Gender		
Male	9 (82%)	13 (93%)
Female	2 (18%)	1 (7%)
Ethnicity		
White	8 (73%)	10 (71%)
Asian	3 (27%)	4 (29%)
Black	0 (0%)	0
Employed		
Yes	9 (82%)	13 (93%)
No	2 (18%)	1 (7%)
Relationship^a^		
Married/civil partner	6 (55%)	7 (54%)
Partner	3 (27%)	6 (46%)
Family member	1 (9%)	0
Highest level of education		
Degree or above	6 (55%)	2 (21%)
A level equiv	1 (9%)	2 (14%)
GCSE equiv	3 (27%)	8 (57%)
No quals	1 (9%)	1 (7%)

*NB:*^a^ Missing relationship data for 1 participant in each phase

^b^ Includes one female ‘birth partner’, a family member of an included woman participant

### Intervention implementation

To minimise contamination across phases midwife care coordinators were identified, and commenced training, immediately prior to Phase 2. Different approaches were taken in the two sites, to accommodate variations in service configuration. In Site 1, interest was sought across both hospital and existing community case-holding midwifery teams, 8 midwives attended 3 initial 2-h training sessions with the Chief Investigator (CI) and research midwife. In Site 2, midwives were identified in consultation with an antenatal service manager from the clinic teams, which covered 2 hospital locations. To accommodate service demands and leave, 4 midwives (2 per hospital) were trained one-to-one in Site 2. All care coordinators received a study manual and were invited to separate training sessions for the ‘Coping Strategies’ toolkit (included in the manual), facilitated by the study health psychologist [DS], which was attended by 5 care coordinators. All women recruited in Phase 2 were allocated a named care coordinator and ‘buddy’. To avoid overburden; no more than 3 women were assigned to each care-coordinator at any one time. In Site 1, it was recognised that several women had existing relationships with midwives not currently acting as care coordinators in the study. In agreement with service managers, additional one to one study training permitted these midwives to take on the role of care coordinator for the woman, if willing. An additional 8 midwives at Site 1, were trained during the period. Nine of the 17 midwives trained in Site 1 and all 4 midwives trained in Site 2 were allocated to women, and all successfully initiated contact after recruitment.

The pattern of antenatal contacts during participant’s entire pregnancies and data for continuity of midwifery carer for women in both phases are summarised in Table 5. The total number of planned antenatal visits and midwife visits for women were similar across both phases. In Phase 2, the number of direct contacts between women and care coordinators (or ‘buddies’) was variable; the median was 2 visits (range: 0–14), equating to 24% (0–74%; median and range) of total planned midwife visits. To enable comparison across both phases, the number of individual midwives seen, per woman, was defined and the midwife seen most frequently identified as ‘lead midwife’. In both phases, the median number of visits with the ‘lead’ midwife was 4, (% median [range]) Phase 1; 37 [18–100] % vs Phase 2 40 [14–84] %). The total number of individual midwives seen by each woman was also similar in both phases. Although data for ‘midwife seen’ was missing for 9% of visits, these findings suggest that the study intervention did not increase continuity of carer, assessed by direct contacts at visits. Intervention logs, supplied at allocation and completed by the care coordinators in Phase 2, captured other implementation data. Of 38 logs issued, 33 were returned (87%), 3 were returned blank and one had no antenatal entries. A median of 3 contacts per woman, (range 1–13) were entered, per log, including face to face, telephone, SMS, and email. Fourteen logs (46%) recorded an explanation of the care coordinator’s role and 12 (40%) a discussion of antenatal and birth care plans; a few reported liaising with multidisciplinary professionals where women required input outside the maternity care team during pregnancy. In logs with entries, 14 women (42%) were documented to have received contact (in-person or by phone) from the care coordinator or buddy after the birth (range 1–4 occasions). Interviews and the research midwife study logs suggest that the intervention logs were often not completed contemporaneously or consistently and may have under-reported intervention-related activities in some cases. The study ‘*WhatsApp’* group included 21 women (55%), during Phase 2. Initially, some participants used the group to introduce themselves and interact with others and the research team. As the study progressed interaction declined, a few users posted regularly, particularly to share information about local support, media reports and websites of interest. The research team also used the group to publicise details of the support sessions, held monthly until December 2019. Despite regular publicity via the research teams and care coordinators the support sessions were not well attended, only 5 (13%) women accessed these in total during Phase 2.

**Table 5:** Antenatal contacts during pregnancy, by study phase

	Phase 1 (Pre intervention)	Phase 2 (Intervention)
***Planned antenatal visits***		
Total antenatal visits	18 (16–24; 5–38)	18 (15–21; 8–31)
Total midwife visits	11 (9–14; 2–26)	11 (9–15; 4–21)
Care coordinator or buddy visits	0	2 (1–6; 0–14)
% Visits saw care coordinator or buddy	0	24 (9–43; 0–74)
‘Lead’ midwife^a^ visits	4 (2–6;1–15)	4 (3–5; 1–16)
% Visits with ‘lead’ midwife^a^	37 (32–51; 18–100)	40 (29–52; 14–84)
Other midwife visits	6 (4–7; 0–13)	6 (4–9; 2–14)
Midwife not recorded	1 (0–1; 0–3)	1 (0–2; 0–5)
Total number of midwives seen	6 (5–7; 1- 12)	6 (4–7; 3–11)
***Unplanned antenatal visits***		
Total visits	2 (1–3: 1–5)	2 (1–5; 1–10)

Data are median (IQR; range), unless stated

NB: Midwife seen was not recorded in notes for 9% of antenatal visits. ^a^ ‘Lead’ midwife was defined as the midwife seen most frequently during planned antenatal visits. If two midwives saw woman for same number of visits ‘lead’ was designated as the first midwife identified. In phase 2, the ‘lead’ midwife for 9 women 4 in site 1 and 5 in site 2 was a midwife other than the care-coordinator or buddy

### Outcomes and acceptability

Clinical outcomes for women participants and their babies are summarised in Table 6. Of the 5 women who withdrew from questionnaire completion before birth, 4 agreed to outcome data being collected from hospital records. Fifty women (96%) had a live birth, one participant experienced a second-trimester fetal death and one baby, born extremely preterm, died in the neonatal period. All adverse events and outcomes affecting participants were reviewed by the Chief Investigator and reported to the Technical Advisory Group, none were related to the research or study intervention. Gestation at birth, labour onset, mode of birth, baby birth weight and length of hospital stay were similar between participants in both phases. Only one participant was still pregnant at the time of the first national lockdown during the COVID-19 pandemic in March 2020, in her postnatal interview limited impacts on care were described (partner was unable to attend hospital appointments and some were held remotely), therefore these data were included in the analysis.

**Table 6:** Postnatal and neonatal outcomes

Characteristics	Phase 1	Phase 2
**Outcome**	*N* = 15	*N* = 37
Miscarriage	0	1 (3%)
Live birth- still living	15 (100%)	35 (94%)
Live birth-neonatal death	0	1 (3%)
**Gestation** Median (range)	38 (36–40)	37 (23–40)
**Labour onset**	*N* = 15	*N* = 36
Spontaneous	3 (20%)	6 (17%)
Induction	7 (47%)	16 (44%)
None (pre-labour CS)	5 (33%)	14 (39%)
**Birth mode**		
Spontaneous	8 (53%)	18 (50%)
Ventouse/forceps	1 (7%)	1 (3%)
Planned Caesarean	3 (20%)	14 (39%)
Unplanned Caesarean	3 (20%)	3 (8%)
**Birthweight (grams):** Median (range)	3020 (2550 – 3620)	2965 (498 – 4280)
**Baby sex**		
Male	9 (60%)	21(58%)
Female	6 (40%)	15 (42%)
**APGAR** (5 min) Median (range)	10 (8 – 10)	9 (4 – 10)
**Length of hospital stay** (days): Median (range)	2 (0 – 10)	2 (0 – 9)
**Feeding** at discharge	*N* = 15	*N* = 34
Breast	10 (67%)	15 (44%)
Mixed	1 (7%)	9 (26%)
Artificial	4 (27%)	10 (29%)
Postnatal and Neonatal Complications		
**Maternal and birth complications**	4 (27%)	10 (27%)
Post-Partum Haemorrhage	1	6
Abnormal vital signs	2	1
Hypertension	1	0
Other	0	4
**NICU admission**	2 (15%)	10 (27%)
Respiratory distress	2	2
Infection	0	3
Prematurity	0	3
Poor feeding	0	1
Other	0	1
**NICU length of stay** (days): Median (range)	6 (2 – 9)	4(1 – 37) ^a^
**Unplanned healthcare contacts**	4	18
Maternal	3	17
Neonatal	1	1

^a^ Data for length of stay for 2 babies was missing

Psychological outcome data for women and partners are presented in supplementary material Figs. S1 and S2, utility values and QALYs are summarised Table S1. The psychological and health status questionnaires and EQ-5D-5L were completed by all women participants at recruitment and those remaining in the study at follow-up. A small number of missing responses to individual questions were identified, but no required question was repeatedly omitted. Two participants did not complete the postnatal questionnaires. None of the psychological outcomes for women, or accrual of QALYs differed notably between phases. Based on the intervention logs, the estimated cost of providing the intervention (including training midwives) was £132/participant. Qualitative interviews explored experiences of care, the intervention, and the research process with 20 women (6 in Phase 1 and 14 in Phase 2, 5 partners and birth partners (1 in Phase 1 and 4 in Phase 2) at 5–6 weeks after birth, and 8 health workers (all were midwives directly or indirectly involved in delivering the intervention, including 7 care coordinators and ‘buddies’ and one specialist midwife with service management responsibility) following completion of Phase 2.

In Phase 2, two main themes were identified from the women’s interviews. Women perceived the intervention as a ‘good idea, but variable practice’ implying delivery was not consistent, and research participation was seen as ‘a worthwhile experience’. Subthemes and example supportive quotes are presented in Table 7. Seeing the same midwife as often as possible during their subsequent pregnancy was consistently identified as important to women, whether they received the study intervention or not. Notably, amongst the women participating pre-intervention, one was offered, and another actively sought antenatal continuity. In both cases women had existing relationships with community midwives from previous pregnancies, who facilitated flexible and additional appointments. Women’s experiences of the intervention were variable; those who had substantial contacts overwhelmingly described positive supportive relationships with their care coordinator or buddy. These were perceived as improving the overall experience of care during the pregnancy (Table 7). Opportunities to build trust with the care coordinator and empathy demonstrated by communicating understanding of emotions and needs during pregnancy were considered key facilitators of a good relationships by women. For others, direct contacts were described as sporadic or few.Several women described follow-up calls and messages received when the care coordinator or buddy was unable to attend appointments which were highly appreciated. Women recognised services pressures, including busy and over-running clinics as important barriers to providing continuity of care.

**Table 7:** Experiences of care and acceptability of research (Women Phase 2): Sample quotations

Theme	Sub theme	Quote
**‘Good idea, variable practice’**	***Feeling supported and cared for***	‘…*but when I first started going, erm, again, I started…it was, like, before I went into the study and before I…before I got under a specific consultant, I was seeing quite a few different people* *And that was then when I went into the study. So I could speak to [care coordinator] then, after that. And she…she then got me back in to see Dr[consultant]and, from that point on then, I was seeing Dr [consultant] more. So I had, like, a lead consultant…* *And, you know, and…and then I had [care coordinator] as someone who, you know, she…she was great, as in, you know, she give me her number to, you know, text me whenever you…’* **Sally**^**a**^
	***Building supportive relationships***	*‘I am aware that [care coordinator] has really taken note of my pregnancy to the point where, you know, she’s seen my baby and she’s got a picture of that. I never have done that in any of my pregnancies. So, that, that is quite nice to know. And actually, I suppose it’s an understanding that it can sometimes mean as much to your midwife as it does to you, and I think you miss that sometimes, but…’* **Louise**^**a**^
		*‘Erm, but the thing I liked about [care coordinator] was…is that unlike the other professionals she spoke to both of us. And she always asked after [partner] and was concerned about him and erm…yeah, and just talked to him as well. And that was, that was something that [partner] and I hadn't really seen before. Erm…’ ****Fiona***^**a**^
	***System needs ‘tweaking’***	*‘but within the community, I don't think I've seen the same midwife twice. And then, my hospital appointments, I know that I had a named, sort of, midwife. Erm, and again, I think that is good, but again, you’ve got holidays. And like, one time, I went in, and I knew, I'd had a message off [care coordinator], she'd texted me…to introduce herself, and say, this is the number if you need anything. Erm, and I'd asked, I said, oh is she here, and they said, oh she's just finished.’* **Madison**^**a**^
		*‘…and I spoke to her a load of times on the phone and it was weird because every time I had an appointment, she either was at [name of unit] when I was here or was on annual leave but I did speak to her a lot and she rang me up quite a lot and we chatted****.’* Patricia**^**a**^
**Research participation: ‘a worthwhile experience’**	***Making a difference***	*‘Yeah. Yeah, I think it helps me personally and then I feel like I’m going to make a difference to somebody else as well because [baby murmurs] the more you find out through this study the…maybe you can do something to help other women maybe deal with it a bit better than what I would have done if you know what I mean****.’ Saheena***^**a**^
		*‘Yeah. Then in that case I feel like it’s… Yeah. For me, personally, it’s been useful. No, I think I just want to say that y-, I know that it can be difficult to get social research approved in the NHS, but I think… Particularly where it’s a sensitive kind of subject, all the ethics of it and all this, that and the other. But I just… I think it’s great that you’re…that the hospital or the trust or whoever have kind of taking the time to do this…’ ****Louise***^**a**^
	***My voice was heard***	*‘Yeah, because you’d never speak to someone, you know, you’d never know, you know, we’ve spoke quite a bit, haven’t we Erm, so, yeah, I…I’m…I’ve found it a benefit, I think it’s good. And being able to give my feedback about how good it’s been really.’ ****Sally***^**a**^

^a^ All names are pseudonyms

Views on the additional components of the intervention offered were more mixed. In Phase 2, all women recalled receiving information about the support group, but most had not attended. Timing and transport were mentioned as barriers by some women. Several women also highlighted concerns around including women at different stages of pregnancy in the same group and others had past negative experiences with pregnancy loss support groups. Overall, women and partners were extremely positive about involvement in research around improving care after stillbirth or neonatal death. Most considered the study questionnaires acceptable, and interviews were particularly welcomed as an opportunity to explore experiences and areas for improvement in care.

The midwives interviewed also recognised the value of continuity, and the potential of the intervention to improve care for women. However, despite considerable efforts care coordinators described difficulties in maintaining the level of contact they wished with their allocated women. Service pressures, shift and roster changes, leave and part-time working were identified as having negative impacts on the midwives’ ability to exert control over their working activities. Telephone calls and text messages were found to be useful and not excessively time consuming by several of the care coordinators, however frustration with inability to maintain direct contact was expressed. Several midwives felt that current organisation of services tended to prioritise completion of care ‘tasks’ and perceived that more radical change in working practices was needed to improve relational care. Changes in service management across both sites occurred during the research and not all managers were perceived to be supportive of the research. Despite the challenges, several midwives felt they had gained personally through involvement in the study. They valued training particularly for improving understanding of women’s and families needed and expressed satisfaction from their enhanced role:

*‘I really enjoyed it.…just job satisfaction as well and it’s like, er, just to make sure they’re alright and like port of call for contact, and to make sure they’ve got follow up appointments…’ (Care Coordinator).*

## Discussion

This was the first UK study to explore the feasibility of implementation and a full-scale trial of a package to improve care and emotional support during pregnancy after stillbirth or neonatal death. Across global settings there have been few trials in this area and none, we are aware of, assessing supportive interventions [29]. The intervention, focussed on improving relational continuity and support through allocation of a named and known midwife care coordinator, access to additional and online support during pregnancy, was successfully implemented in two NHS maternity providers in North-West England. Near-target recruitment and participant retention above 75%, demonstrated women’s willingness to participate in research in this sensitive area. Women, partners and midwives involved were overwhelmingly supportive of enhanced continuity to improve care and positive about the research. Data collection methods proved generally acceptable, with interviews particularly valued by participants as an opportunity for sharing experiences and ideas for developing services further.

### Strengths and limitations

This study was conducted in two maternity care organisations serving culturally diverse populations including areas of significant socio-economic deprivation in Northwest England. Reflecting the setting, 40% of study participants described their ethnicity as Asian or Black. Asian and Black women and those living in deprived areas are significantly more likely to experience perinatal death [30] and are often under-represented in research, particularly surrounding maternity experiences [31]. Nevertheless, a number of potential participants were excluded by language barriers, as it was not possible to provide translation within the scope of this feasibility study. Women who do not speak English are often disproportionately affected by adverse outcomes and should be prioritised in future studies.

### Interpretation

Consistent evidence supports the importance of specific emotional and psychological support from health professionals for women and families during pregnancy after stillbirth or neonatal death [7, 32]. Despite prioritisation of research by both health professionals and parents [33], few studies have explored development or evaluation of new antenatal care pathways or services [34]. The current study did not aim to assess the impact of the intervention on outcomes; however, the psychological and health status measures reflect previous studies suggesting negative psychological state decreasing as pregnancy progresses [5]. The study intervention was co-designed for maximum flexibility and practicality of implementation alongside existing services. The care co-ordination model, developed in nursing for chronic or complex conditions, which emphasised relational continuity and connections to navigate multidisciplinary care, often required during high-risk pregnancy was adopted as a basis [35]. Previous studies to increase midwifery continuity, mainly in low-risk pregnancy, have focused on case-holding models which required significant workforce flexibility including 24-h on call [12]. These models often proved difficult to sustain beyond research or small-scale contexts in the UK [36, 37]. The role of care- coordinator also required changes in practice from the midwives, although they were not asked to be on call for births, most volunteered to be involved and were highly invested in delivering the intervention as planned. A relatively short duration intervention training session, which could be delivered in groups or individually, allowed additional midwives to take on the role of care co-ordinator as the intervention period progressed. This facilitated participation by women with existing relationships with midwives in site 1 and maximised intervention uptake in this site. When consistent contacts enabled woman-midwife relationships to be built, these findings reflected previous evidence of positive effect on both women’s experiences [38] and midwives’ job satisfaction [39].

Although no target related to continuity for antenatal visits was set, no overall increase was observed for direct contacts with the care coordinator or lead midwife during the intervention phase. The finding that several women receiving ‘usual care’ were offered increased continuity by their community midwives or made specific requests to see the same midwife may have contributed to the lack of difference we observed. However, wider service and organisational factors are also likely to have impacted and our data highlight fragility of the changes made when key personnel were absent, or services were stretched. In a recent evaluation of implementation of continuity of midwifery care in Scotland, McInnes et al. confirmed the importance of behaviours of the entire multidisciplinary team, across all levels and beyond those directly involved, particularly during early stages of introducing complex interventions in pre-existing care structures [37]. Changes in practice leadership in the research sites could also have acted as a barrier to the intervention being accommodated within the traditional high-risk model of care here as new managers were not previously involved and, potentially, less invested in the research. It is noteworthy that our assessment of continuity did not capture remote contacts including phone and text, which were demonstrated to enhance relationships between midwives and women. Additional support components of the intervention, including the monthly support group were not widely taken up. Although no previous UK research has focussed specifically on professional or peer support in subsequent pregnancy after perinatal death, parents previously identified contact with others with shared experiences as potentially helpful in addressing the unique challenges of this situation [8]. In person support groups, connecting women and partners had beneficial effects on experiences of pregnancy in previous studies in US, Canada and Australia [40, 41]. Prior relationships with the facilitators, who were midwives/nurses also providing care for women in pregnancy, often encouraged attendance at these groups. Conscious of risk overburden on clinical staff delivering the intervention in the research sites, the research team organised and facilitated groups for this study. Uptake of support groups may have improved if the care coordinators delivering care and known to women had been in attendance.

The NHS England Maternity Transformation Programme, which commenced after this research began, has accelerated deployment of continuity of carer pathways in NHS maternity services. Some Local Maternity Systems, tasked with implementation, are actively targeting women in specific ‘vulnerable’ groups including, those in pregnancies after perinatal death as a priority group for roll-out of continuity of carer pathways. This policy shift precludes a full-scale UK trial of our package. Our study provided insights around managing change, including the importance of the process as well as focus on outcomes or targets, such as incorporating elements of behavioural change theory to promote and sustain performance. Engaging clinical leaders is crucial, particularly in the first phase of changes when a new model co-exists with established ways of working. Starting the process incrementally with volunteer midwives already committed to continuity acting as ‘change champions’ may enhance embedding the model and motivating more resistant colleagues to participate [42].

## Conclusion

Women, partners and birth partners were willing to participate in research to evaluate interventions to improve care in pregnancy after stillbirth or neonatal death. Continuity of midwifery care during pregnancy offers substantial opportunities to improve emotional support and improve experiences for women and families. However, individual and organisational barriers affected consistency of implementation of the current intervention which could be addressed with refinement of the change management process. Nevertheless, learning from implementing and evaluating this intervention provides key insights to the behavioural, cultural and systems changes needed to realise the vision of ‘Better Births’ which reflects increasing understanding of the importance of more personalised, compassionate and woman-centred maternity care to improve outcomes [17].

## Supplementary Information

**Additional file 1: Figure S1.** Psychological outcomes (Women). **Figure S2.** Psychological outcomes (Partners). **Table S1.** Summary of utility values derived from the EQ-5D-5L for women and partners at baseline (recruitment), follow up (late pregnancy) and postnatal (4-6 weeks) post birth.

## Data Availability

The datasets generated and analysed during the current study are not publicly available due arrangements specified in ethics approval, but anonymised data is available from the corresponding author on reasonable request.
