# Supplementary material for: Better maternity care pathways in pregnancies after stillbirth or neonatal death: a feasibility study
Source: BMC Pregnancy Childbirth. 2022 Aug 10;22:634. doi: 10.1186/s12884-022-04925-3 (PMC9363262; doi:10.1186/s12884-022-04925-3)
Supplement: Supplementary file 1 — Additional file 1: Figure S1. Psychological outcomes (Women). Figure S2. Psychological outcomes (Partners). Table S1. Summary of utility values derived from the EQ-5D-5L for women and partners at baseline (recruitment), follow up (late pregnancy) and postnatal (4-6 weeks) post birth. [file 12884_2022_4925_MOESM1_ESM.docx]

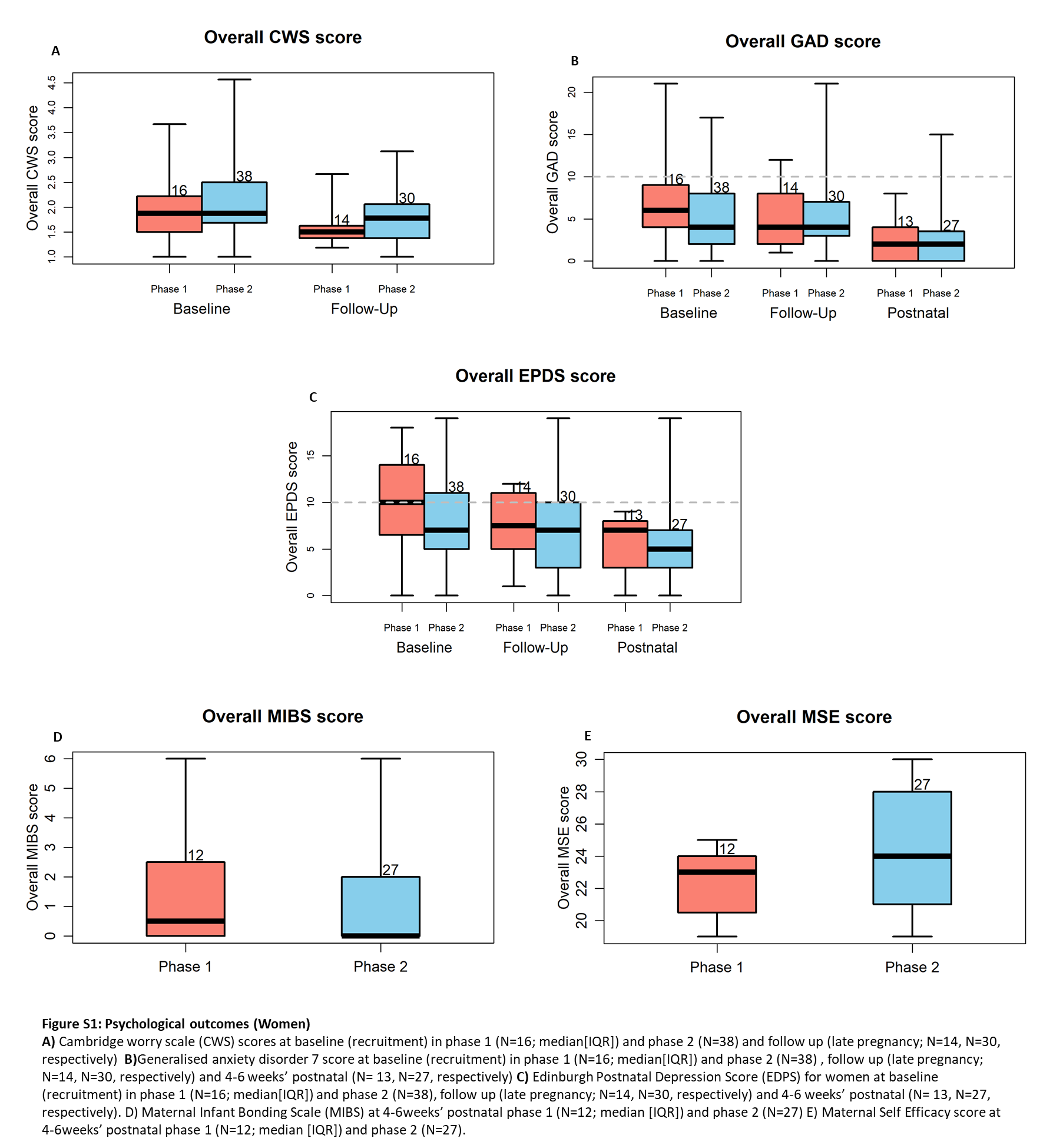


**Figure S1: Psychological outcomes (Women)**

**A)** Cambridge worry scale (CWS) scores at baseline (recruitment) in phase 1 (N=16; median[IQR]) and phase 2 (N=38) and follow up (late pregnancy; N=14, N=30, respectively) **B)**Generalised anxiety disorder 7 score at baseline (recruitment) in phase 1 (N=16; median[IQR]) and phase 2 (N=38) , follow up (late pregnancy; N=14, N=30, respectively) and 4-6 weeks’ postnatal (N= 13, N=27, respectively) **C)** Edinburgh Postnatal Depression Score (EDPS) for women at baseline (recruitment) in phase 1 (N=16; median[IQR]) and phase 2 (N=38), follow up (late pregnancy; N=14, N=30, respectively) and 4-6 weeks’ postnatal (N= 13, N=27, respectively). D) Maternal Infant Bonding Scale (MIBS) at 4-6weeks’ postnatal phase 1 (N=12; median [IQR]) and phase 2 (N=27) E) Maternal Self Efficacy score at 4-6weeks’ postnatal phase 1 (N=12; median [IQR]) and phase 2 (N=27).


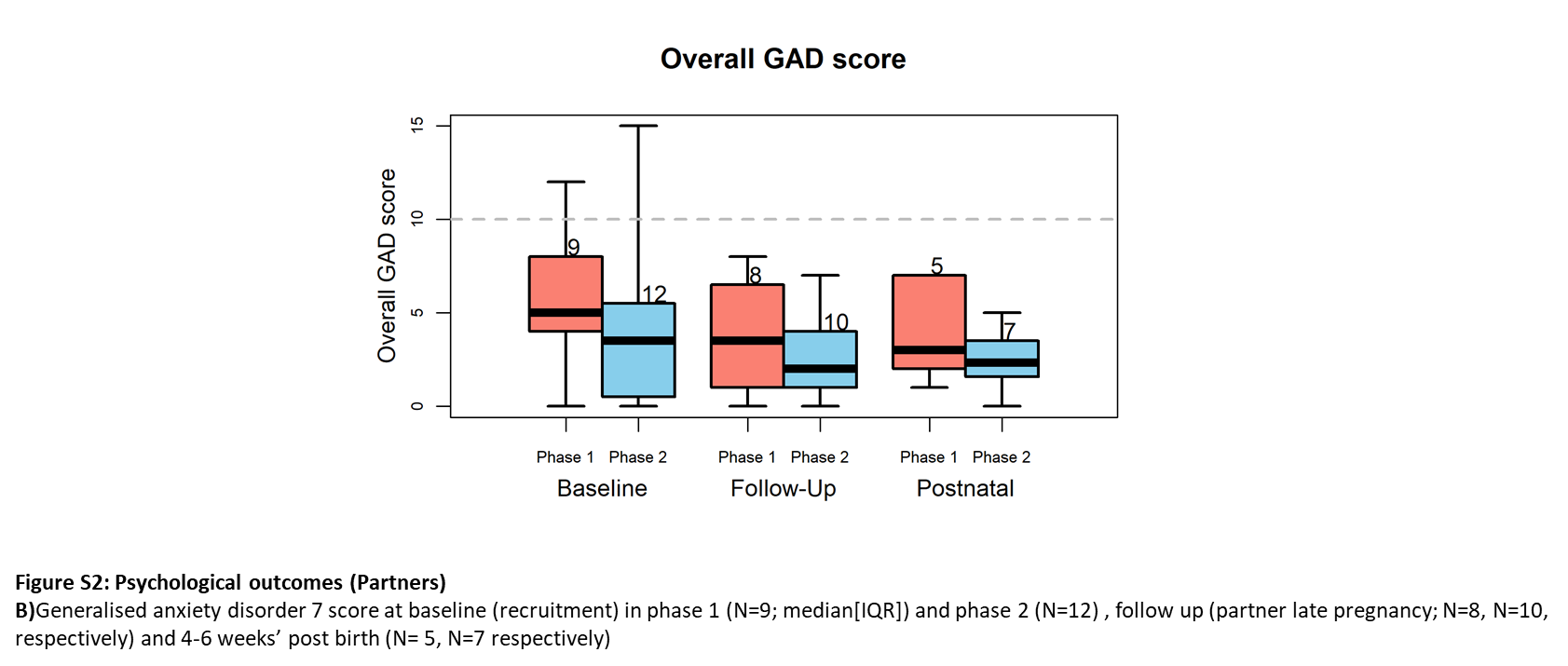


**Figure S2: Psychological outcomes (Partners)**

Generalised anxiety disorder (GAD)-7 score for partners at baseline (recruitment) in phase 1 (N=9; median [IQR]) and phase 2 (N=12) , follow up (partner late pregnancy; N=8, N=10, respectively) and 4-6 weeks’ after birth (N= 5, N=7 respectively)

**Table S1.** Summary of utility values derived from the EQ-5D-5L for women and partners at baseline (recruitment), follow up (late pregnancy) and postnatal (4-6 weeks) post birth

|  | **Women** | |
| --- | --- | --- |
|  | **Phase 1(n=16)** | **Phase 2(n=38)** |
| Utility at Time 1 (baseline) Mean (95% CI) | 0.72 (0.60, 0.85)  n=16 | 0.81 (0.77, 0.86)  n=38 |
| Utility at Time 2 (follow-up) | 0.76 (0.65, 0.88)  n=14 | 0.70 (0.64, 0.81)  n=29 |
| Utility at Time 3 (postnatal) | 0.88 (0.82, 0.95)  n=13 | 0.86 (0.81, 0.91)  n=27 |
| QALYs (baseline to postnatal) | 0.37 (0.32, 0.43)  n=13 | 0.38 (0.35, 0.41)  n=25 |
| Net QALYs (95% CI) | 0.008 (-0.05 to 0.07) n=38 | |
|  | **Partners** | |
|  | **Phase 1(n=9)** | **Phase 2(n=13)** |
| Utility at Time 1 (baseline) | 0.87 (0.80, 0.94)  n=9 | 0.89 (0.80, 0.99)  n=12 |
| Utility at Time 2 (follow-up) | 0.92 (0.86, 0.98)  n=8 | 0.94 (0.87, 0.99)  n=10 |
| Utility at Time 3 (postnatal) | 0.89 (0.81, 0.97)  n=5 | 0.85 (0.69, 1.0)  n=7 |
| QALYs (baseline to follow-up) | 0.50 (0.43, 0.56)  n=5 | 0.45 (0.37, 0.53)  n=6 |
| Net QALYs (95% CI) | -0.04 (-0.14 to 0.05) n=11 | |

**Note:** Although at Time 1 the phase 2 group had higher utility values than the phase 1 group, over the whole follow-up period the groups of women were similar in terms of QALYs. For the partners, those in the phase 1 group accrued slightly more QALYs over the course of the study, however this was based on only 11 individuals.
